# Ebola virus VP35 induces high-level production of recombinant TPL-2–ABIN-2–NF-κB1 p105 complex in co-transfected HEK-293 cells

**DOI:** 10.1042/BJ20121873

**Published:** 2013-05-10

**Authors:** Thorsten Gantke, Sabrina Boussouf, Julia Janzen, Nicholas A. Morrice, Steven Howell, Elke Mühlberger, Steven C. Ley

**Affiliations:** *Division of Immune Cell Biology, MRC National Institute for Medical Research, Mill Hill, London NW7 1AA, U.K.; †Beatson Institute for Cancer Research, Bearsden, Glasgow G61 1BD, U.K.; ‡Division of Molecular Structure, MRC National Institute for Medical Research, Mill Hill, London NW7 1AA, U.K.; §Department of Microbiology, School of Medicine and National Emerging Infectious Disease Laboratories, Boston University, Boston, MA 02118, U.S.A.

**Keywords:** A20-binding inhibitor of nuclear factor κB 2 (ABIN-2), cancer Osaka thyroid (COT), nuclear factor κB 1 (NF-κB1), double-stranded-RNA-dependent protein kinase (PKR), tumour progression locus 2 (TPL-2), virus protein 35 (VP35), ABIN-2, A20-binding inhibitor of nuclear factor κB 2, BMDM, bone-marrow-derived macrophage, DM, decyl β-D-maltopyranoside, DTT, dithiothreitol, ERK, extracellular-signal-regulated kinase, GST, glutathione transferase, HA, haemagglutinin, HEK, human embryonic kidney, Hsp, heat-shock protein, IκB, inhibitor of NF-κB, IKK, IκB kinase, MAPK, mitogen-activated protein kinase, MBP, myelin basic protein, MKK, MAPK kinase, NF-κB, nuclear factor κB, PKR, double-stranded RNA-dependent protein kinase, TBK1, TANK [TRAF (tumour-necrosis-factor-receptor-associated factor)-associated NF-κB activator]-binding kinase 1, TCEP, tris-(2-carboxyethyl)phosphine, TNF, tumour necrosis factor, TPL-2, tumour progression locus 2, VP35, virus protein 35

## Abstract

Activation of PKR (double-stranded-RNA-dependent protein kinase) by DNA plasmids decreases translation, and limits the amount of recombinant protein produced by transiently transfected HEK (human embryonic kidney)-293 cells. Co-expression with Ebola virus VP35 (virus protein 35), which blocked plasmid activation of PKR, substantially increased production of recombinant TPL-2 (tumour progression locus 2)–ABIN-2 [A20-binding inhibitor of NF-κB (nuclear factor κB) 2]–NF-κB1 p105 complex. VP35 also increased expression of other co-transfected proteins, suggesting that VP35 could be employed generally to boost recombinant protein production by HEK-293 cells.

## INTRODUCTION

TPL-2 (tumour progression locus 2) [also known as MAP3K8 (mitogen-activated protein kinase kinase kinase 8) and COT (cancer Osaka thyroid)] mediates activation of ERK (extracellular-signal-regulated kinase) 1 and 2 MAPKs (mitogen-activated protein kinases) by Toll-like receptors and the receptors for TNF (tumour necrosis factor) and interleukin 1β in macrophages [1]. Lipopolysaccharide induction of TNF *in vivo* and in cultured macrophages is dependent on TPL-2 expression [2]. Consequently, TPL-2 has attracted considerable attention as a potential target for the development of small-molecule inhibitors to treat TNF-driven inflammatory diseases, including rheumatoid arthritis, Crohn's disease and psoriasis [3,4].

In unstimulated cells, TPL-2 is stoichiometrically associated with NF-κB (nuclear factor κB) 1 p105 and the ubiquitin-binding protein ABIN-2 (A20-binding inhibitor of NF-κB2), which are both required to maintain TPL-2 protein stability [5–9]. Direct binding of NF-κB1 p105 to the TPL-2 kinase domain also negatively regulates TPL-2 MKK (MAPK kinase) 1/2 activity by blocking access of MKK1/2 to its active site [5,8]. TPL-2 phosphorylation of MKK1/2 and activation of ERK1/2 MAPK signalling consequently requires TPL-2 release from NF-κB1 p105, which is triggered by IKK [IκB (inhibitor of NF-κB) kinase]-induced proteolysis of NF-κB1 p105 by the proteasome following agonist stimulation [10,11].

We have recently made an unexpected discovery about how TPL-2 regulates TNF production by macrophages. Analyses of *Nfkb1*^SSAA/SSAA^ macrophages, in which the IKK target serine residues on NF-κB1 p105 are mutated to alanine, revealed that TPL-2 regulates TNF production independently of IKK-induced NF-κB1 p105 proteolysis and ERK1/2 activation, while still associated with NF-κB1 p105 and ABIN-2 [12]. These data indicate that the TPL-2–ABIN-2–NF-κB1 p105 complex phosphorylates substrates other than MKK1/2 to stimulate TNF production, and that small-molecule inhibitors should target this complex to block TNF production. However, no method is currently available to express and purify the catalytically active TPL-2 signalling complex.

In the present study, a novel methodology to produce milligram quantities of highly pure catalytically active recombinant TPL-2–ABIN-2–NF-κB1 p105 complex in transiently transfected mammalian HEK (human embryonic kidney)-293 cells was developed, which is based on co-expression of Ebola virus VP35 (virus protein 35) to antagonize transfection-induced inhibition of translation. VP35 co-transfection increased protein expression of recombinant TPL-2 complex more than 10-fold. Importantly, we found evidence to suggest that this approach could be readily adapted to boost the expression of any recombinant cytosolic or membrane-bound protein.

## EXPERIMENTAL

### Cell culture

Adherent HEK-293 cells (QBI293A cells, Quantum Biotechnologies) were maintained as described previously [13]. Cells were adapted to growth in suspension using Pro293s-CDM medium (Lonza), supplemented with 1.5% fetal bovine serum, 2 mM l-glutamine, 50 units/ml penicillin and 50 units/ml streptomycin, and were cultured to between 1.5×10^6^ and 4.0×10^6^ cells/ml in 1-litre spinner flasks (Techne). BMDMs (bone-marrow-derived macrophages) were generated from C57BL/6 mice as described previously [10]. All procedures described in the present study involving animals were done in accordance with U.K. Home Office regulations.

### Expression plasmids and antibodies

Expression plasmids encoding His_6_-tagged TPL-2 and TPL-2^D270A^ (NCBI RefSeq NP_001231063.1), FLAG-tagged human TPL-2 and TPL-2^30–397^, and C-terminally StrepII-tagged ABIN-2 (NCBI RefSeq NP_077285.3) were generated by PCR amplification and subcloning into the pCDNA3 vector (Life Technologies). The pCDNA3-3xHA-IKK-2 expression construct was generated by subcloning from pRc-β-actin-3xHA-IKK-2 (provided by Professor Michael Karin, University of California San Diego, San Diego, CA, U.S.A.). Plasmids encoding Bcl-2 (pMT2), CD40 (pSRalpha), Lck (pCDNA3), HA (haemagglutinin)-tagged NF-κB1 p105 (pCDNA3), Myc–His–TBK1 {TANK [TRAF (TNF-receptor-associated factor)-associated NF-κB activator]-binding kinase 1} (pCDNA3), and HA–VP35 of the Ebola virus species *Zaire ebolavirus* (Mayinga isolate) [pCDNA3.1/myc-His(−)A] have been described previously [14–18]. Coding sequences of all expression plasmids were verified by DNA sequencing.

Antibodies against TPL-2 (sc-1717 and sc-720), RelA (sc-372-G), c-Rel (sc-71), RelB (sc-226), Bcl-2 (sc-509), CD40 (sc-9096), MKK1/2 (sc-81504), Hsp (heat-shock protein) 70 (sc-24), Hsp90α/β (sc-7947) and actin (sc-1615) were purchased from Santa Cruz Biotechnology. Antibodies against pThr^446^-PKR (double-stranded-RNA-dependent protein kinase) (ab32036) and PKR (ab32506) were purchased from Abcam. Antibodies against NF-κB1 p105/p50 (#3035), pSer^932^-NF-κB1 p105 (#4806), Lck (#2752), pSer^221^-MKK1/2 (#2338) and the Myc-tag (#2276) were from Cell Signaling Technology. Anti-FLAG (F7425) and anti-HA (#11867423001) antibodies were from Sigma–Aldrich and Roche respectively. TAT-1 anti-α-tubulin antibody was provided by Professor Keith Gull (University of Oxford, Oxford, U.K.). ABIN-2, pSer^400^-TPL-2 and TPL-2 (TSP3) antibodies have been described previously [7,19,20]. Band intensities were measured by laser densitometry using a GS-800 densitometer (Bio-Rad Laboratories).

### Transient transfection and cell lysis

Recombinant His_6_- or FLAG-tagged TPL-2–ABIN-2–StrepII–HA–p105 complex was expressed in adherent HEK-293 cells as described previously [21]. At 48 h after transfection, cells were lysed in 50 mM Tris/HCl (pH 7.5), 1% SDS, 150 mM NaCl, 2 mM DTT (dithiothreitol), 2 mM EDTA, 10 mM NaF, 1 mM sodium pyrophosphate, 100 nM okadaic acid and 10 mM 2-glycerophosphate plus Complete™ Protease Inhibitor Cocktail (Roche) and analysed by immunoblotting.

For expression of recombinant TPL-2 complex and FLAG–TPL-2^30–397^ in non-adherent HEK-293 cells, cells were pelleted by centrifugation, and resuspended at a density of 4.0×10^6^ cells/ml in standard culture medium, before addition of DNA complexed with linear polyethyleneimine (25 kDa) at a ratio of 3:1 (w/w) to a final concentration of 2 μg of DNA/ml (including 0.25 μg of HA–VP35 plasmid/ml). Cell density was adjusted to 2.0×10^6^ cells/ml after 5 h and cells were lysed after 72 h. For subsequent purification, cells were lysed in buffer A {50 mM Tris/HCl (pH 7.5), 0.5% Igepal CA-630, 150 mM NaCl, 10 mM imidazole, 10 mM NaF, 1 mM sodium pyrophosphate, 10 mM 2-glycerophosphate, 0.5 mM TCEP [tris-(2-carboxyethyl)phosphine], 10% glycerol and protease inhibitors}. FLAG–TPL-2^30–397^ was purified after lysis in 50 mM Tris/HCl (pH 7.5), 1% Triton X-100, 150 mM NaCl, 1 mM EDTA, 10 mM NaF, 1 mM sodium pyrophosphate, 10 mM 2-glycerophosphate, 2 mM DTT, 100 nM okadaic acid and 10% glycerol, supplemented with protease inhibitors.

### Protein purification and analyses

Recombinant His_6_–TPL-2–ABIN-2–StrepII–HA–p105 complex was subjected to three-step affinity purification. For this, centrifuged lysates were incubated with Ni-NTA (Ni^2+^-nitrilotriacetate)–agarose (Qiagen) for 60 min, washed in DM wash buffer [50 mM Tris/HCl (pH 7.5), 1.8 mM decyl β-D-maltopyranoside (DM), 150 mM NaCl, 10 mM imidazole, 10 mM NaF, 1 mM sodium pyrophosphate, 10 mM 2-glycerophosphate, 0.5 mM TCEP and 10% glycerol supplemented with protease inhibitors], before elution with 200 mM imidazole. After adjusting EDTA to 1 mM, eluates were incubated with StrepTactin Sepharose (GE Healthcare) for 60 min, washed with imidazole-free DM wash buffer. Bound protein was eluted by the addition of 2.5 mM desthiobiotin and then loaded on to an Ni^2+^-charged HisTrap HP column (GE Healthcare), washed with 50 mM Tris/HCl (pH 7.5), 1.8 mM DM, 150 mM NaCl, 10 mM imidazole, 0.5 mM TCEP and 10% glycerol before elution in 200 mM imidazole. Sample purity was quantified by measuring the IR fluorescence of Coomassie Brilliant Blue-stained protein gels (Odyssey IR imaging system, LI-COR Biosciences). MS analysis of purified protein was performed as described previously [22].

Recombinant FLAG–TPL-2^30–397^ and FLAG–TPL-2–ABIN-2–StrepII–HA–p105 complex was purified by incubating cleared lysates with ANTI-FLAG® M2 Affinity Gel (Sigma–Aldrich) for 60 min, followed by extensive washing with lysis buffer and elution with 0.2 mg/ml 3×FLAG peptide (Sigma–Aldrich) in DM buffer.

Gel-filtration experiments were performed using a Superose 6 10/300 GL column (GE Healthcare). For analysis of endogenous TPL-2 complexes, BMDMs were lysed in buffer A, and 1.25 mg of cleared lysate was loaded on to a Superose 6 10/300 GL column, calibrated using a mixture of molecular mass marker proteins (MWGF1000, Sigma–Aldrich).

### Kinase assays

Catalytic activities of recombinant TPL-2 complex and FLAG–TPL-2^30–397^ towards MBP (myelin basic protein) (Sigma–Aldrich) and GST (glutathione transferase)–MKK1 were determined *in vitro* using 10 nM kinase and 3.5 or 0.1 μM substrate respectively. Assays were performed in kinase buffer, supplemented with 1 mM ATP with or without 10 μM C34 TPL-2 inhibitor, in the presence (MBP) or absence (GST–MKK1) of 0.05 μCi/μl [γ-^32^P]ATP for 30 min at 30°C. Reactions were stopped by boiling in SDS sample buffer and then resolved by SDS/PAGE for subsequent analysis of MBP and GST–MKK1 phosphorylation by autoradiography and immunoblotting respectively.

## RESULTS AND DISCUSSION

### VP35-mediated enhancement of protein expression

Owing to its critical role in regulating the production of TNF by macrophages in innate immune responses, TPL-2 has attracted attention as a potential drug target for TNF-dependent autoimmune diseases [23]. However, despite considerable effort by the pharmaceutical industry [23], no TPL-2 inhibitors have yet entered pre-clinical development. As full-length TPL-2 is largely insoluble when expressed alone, inhibitor screens have used a truncated form of TPL-2 (TPL-2^30–397^) with improved solubility [24,25]. However, purification of TPL-2^30–397^ from baculovirus-infected insect cells achieves only 40% purity, with a substantial fraction aggregated and catalytically inactive. Consequently, there is a need for alternative strategies to produce soluble and catalytically active TPL-2 kinase, preferably in its native full-length conformation. In the present study, we developed a novel method to produce substantial amounts of purified soluble full-length TPL-2 associated with its cellular binding partners ABIN-2 and NF-κB1 p105.

The mammalian HEK-293 cell line is widely used as an expression host for the transient expression of recombinant proteins by transient transfection, as well as for commercial production of recombinant therapeutics, because of its ability to provide a close to physiological environment for exogenously expressed mammalian proteins [26–28]. However, protein yields are usually much lower than in most commonly used non-mammalian expression hosts, such as *Escherichia coli*, yeast and baculovirus-infected insect cells [29].

**Figure 1:**
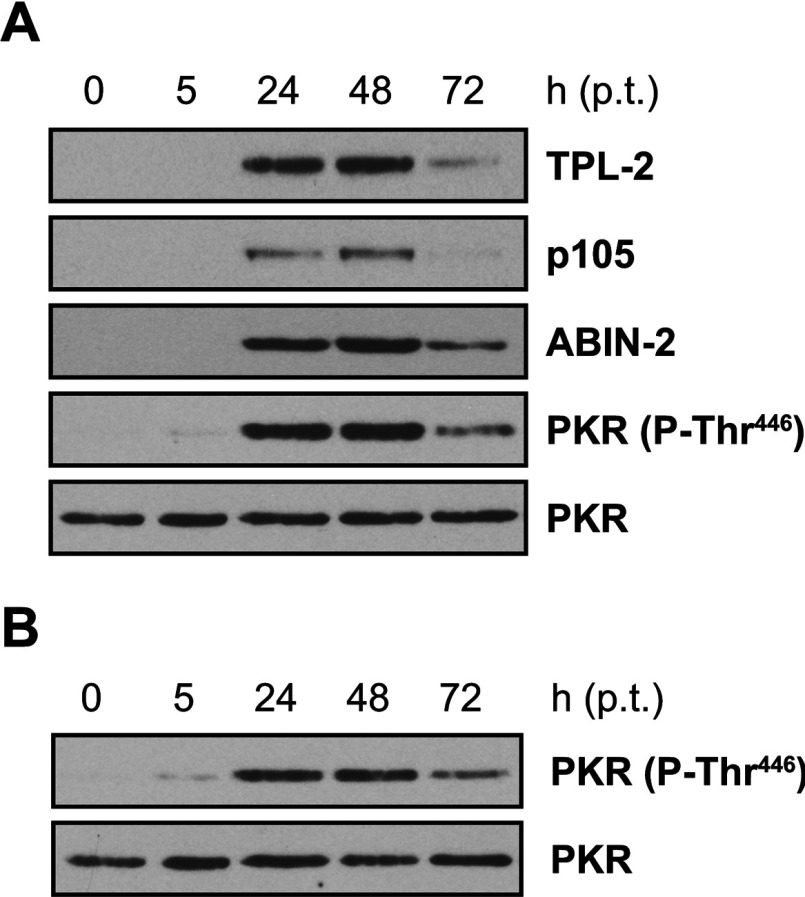
Transient transfection of HEK-293 cells activates PKR (**A**) HEK-293 cells were transiently transfected with expression plasmids encoding His_6_–TPL-2, ABIN-2–StrepII and HA–p105. Protein expression levels and PKR autophosphorylation on Thr^446^ were monitored by immunoblotting of cell lysates prepared 5, 24, 48 and 72 h posttransfection (p.t.). (**B**) Immunoblot analysis of PKR autophosphorylation on Thr^446^ in lysates of cells transiently transfected with empty control plasmid only.

As shown previously, transfection of HEK-293 cells with DNA plasmids activated intracellular antiviral signalling pathways that restricted protein synthesis through activation of PKR, which inhibits translation by phosphorylating the α-subunit of eIF2 (eukaryotic translation initiation factor 2) [15,30] (Figure 1A). Transfection-induced PKR activation was independent of recombinant protein expression, as empty vector had the same effect (Figure 1B). This suggested that PKR-mediated inhibition of translation may limit the amount of recombinant protein that can be produced in HEK-293 cells by transient transfection.

Viruses have evolved elaborate mechanisms to subvert PKR activation in infected cells [30]. For example, the VP35 protein of Ebola virus binds to double-stranded RNA and antagonizes PKR activation following viral infection [31]. Importantly, VP35 has been shown to also inhibit PKR activation in cells transiently transfected with DNA plasmids [15]. This raised the possibility that VP35 could be employed to boost the transient expression of recombinant proteins in HEK-293 cells.

**Figure 2:**
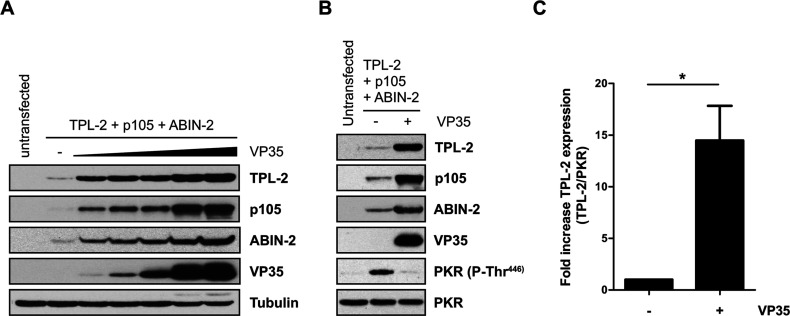
VP35 enhances protein expression of co-transfected TPL-2, ABIN-2 and NF-κB1 p105 (**A**) HEK-293 cells were transiently transfected with expression vectors encoding His_6_–TPL-2, ABIN-2–StrepII and HA–p105 (1 μg each) with or without increasing concentrations of expression plasmid encoding HA–VP35 (0.05–0.8 μg) or were left untransfected. After 48 h of culture, cell lysates were immunoblotted. (**B**) HEK-293 cells were transiently transfected with expression plasmids encoding each of the TPL-2 complex components (as in **A**) with or without 0.8 μg of VP35 plasmid. Protein expression levels and PKR activation were analysed by immunoblotting. (**C**) Laser densitometric quantification of fold increases of His_6_–TPL-2 expression upon HA–VP35 co-transfection in six independent experiments performed as in (**B**). Results are mean±S.D. increases (*P=0.01; Student's paired *t* test).

To test this, HEK-293 cells were transiently transfected with plasmids encoding TPL-2, ABIN-2 and NF-κB1 p105 together with increasing amounts of an expression plasmid encoding Ebola virus VP35 or empty vector. At 48 h after transfection, protein expression levels and PKR activation status were monitored by immunoblotting. Strikingly, VP35 induced a dose-dependent increase in the levels of each of the recombinant proteins (Figure 2A). As expected, VP35 expression substantially decreased PKR activation (Figure 2B). Quantification of six separate experiments demonstrated that co-transfection of VP35 plasmid induced a mean 15-fold increase in TPL-2 expression (Figure 2C). VP35 co-expression might therefore allow the isolation of significantly increased amounts of recombinant TPL-2 kinase complex from transiently transfected HEK-293 cells.

### Purification of recombinant TPL-2–ABIN-2–NF-κB1 p105 complex from HEK-293 cells co-expressing VP35

**Figure 3:**
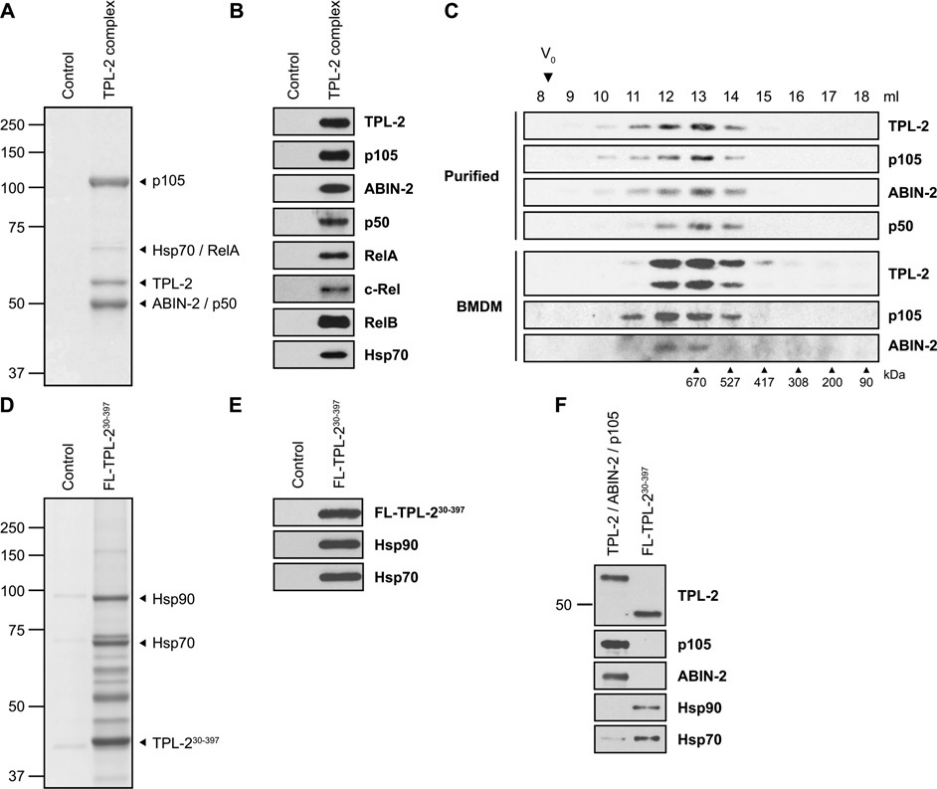
Affinity purification of TPL-2–ABIN-2–NF-κB1 p105 complex HEK-293 cells were transiently transfected with expression vectors encoding His_6_–TPL-2, ABIN-2–StrepII and HA–p105 or empty vector together with HA–VP35 vector. After 72 h, the TPL-2 complex was subjected to three-step affinity purification. Protein eluates were resolved by SDS/PAGE and analysed by Coomassie Brilliant Blue G-250 staining (**A**) or immunoblotting (**B**). Arrowheads in (**A**) indicate the position of the most abundant proteins identified by MS analysis. (**C**) Purified recombinant TPL-2–ABIN-2–NF-κB1 p105 complex was resolved by gel filtration, using a Superose 6 10/300 GL column. Eluate fractions (1 ml) were immunoblotted. V_0_, void volume. (**D**) HEK-293 cells were co-transfected with vectors encoding FLAG–TPL-2^30–397^ or with no insert, plus HA–VP35 vector. Cell lysates (72 h) were immunoprecipitated with anti-FLAG antibody and FLAG peptide-eluted proteins were resolved by SDS/PAGE, and analysed by Coomassie Brilliant Blue G-250 staining and immunoblotting (**E**). (**F**) Purified recombinant TPL-2–ABIN-2–NF-κB1 p105 complex and FLAG-TPL-2^30–397^ were immunoblotted. Molecular masses (kDa) of marker proteins are indicated. FL, FLAG.

It was next tested whether recombinant TPL-2–ABIN-2–NF-κB1 p105 complex could be efficiently purified from non-adherent HEK-293 cells co-expressing VP35. A three-step affinity purification protocol was developed that involved sequential purification of His_6_–TPL-2 and ABIN-2–StrepII via their affinity tags. Analysis of eluates by SDS/PAGE and Coomassie Brilliant Blue staining revealed three major bands migrating at approximately 110, 60 and 50 kDa (Figure 3A), the main constituents of which were subsequently confirmed as NF-κB1 p105, TPL-2 and ABIN-2 respectively by MS (Table 1). MS analysis also indicated specific co-purification of p50, RelA, c-Rel, RelB and the molecular chaperone Hsp70, but not VP35, with recombinant TPL-2 complex, which was confirmed by immunoblotting (Figure 3B). The purity of isolated TPL-2–ABIN-2–NF-κB1 p105 complex was >90%, with a yield of over 1 mg of purified complex per litre of cultured non-adherent HEK-293 cells.

**Table 1:** MS analysis of purified protein complexes

Protein	Gene name	Molecular mass (kDa)	Coverage (%)	Number of peptides
TPL-2 complex				
TPL-2	MAP3K8	53.9*	84	29
NFκB1 p105/p50	NFKB1	106.9*/49.2†	72	71
ABIN-2	TNIP2	49.8*	66	35
Hsp70	HSPA1A	70.0	61	30
RelA (p65)	RELA	60.2	36	13
c-Rel	REL	68.5	17	8
RelB	RELB	62.1	8	3
TPL-2^30–397^				
TPL-2	MAP3K8	42.9*	70	100
Hsp90α/β	HSP90AA1	85.6	57	97
	HSP90AB1	83.3	56	53
Hsp70	HSPA1A	70.0	60	105

*Molecular mass of recombinant protein.

†Molecular mass calculated according to Ciechanover et al. [41].

Co-isolation of Rel proteins was probably due to specific interaction with NF-κB1 p105, which functions as an IκB protein [32], and was consistent with earlier reports showing co-precipitation of Rel proteins with TPL-2 and ABIN-2 [6,7,33]. Hsps have been reported to associate with aggregated FLAG-tagged TPL-2^30–397^ when purified from insect cells [25]. Association of small amounts of Hsp70 with purified TPL-2–ABIN-2–NF-κB1 p105 complex thus raised the possibility that the recombinant complex might have been, at least partially, aggregated. However, gel-filtration experiments indicated that TPL-2 complexes were soluble and not aggregated. TPL-2, ABIN-2 and NF-κB1 p105 co-eluted as one peak together with NF-κB1 p50, with an approximate molecular mass of 670 kDa, and no complex was detected in the void volume. Furthermore, analysis of total cell lysates of BMDMs by gel filtration demonstrated that endogenous TPL-2, ABIN-2 and NF-κB1 p105 were also present in complexes of approximately 670 kDa (Figure 3C), consistent with the previously reported molecular mass of NF-κB1 p105-containing complexes [34].

FLAG–TPL-2^30–397^ purified from transiently transfected HEK-293 cells co-expressing VP35 was of low purity (~21%) and stoichiometrically associated with Hsp70 and Hsp90 (Figures 3D and 3E, and Table 1). Immunoblotting revealed that significantly more Hsp70 bound to TPL-2^30–397^ than to the purified TPL-2–ABIN-2–NF-κB1 p105 complex, whereas Hsp90 only detectably associated with TPL-2^30–397^ (Figure 3F).

Together, these findings suggest that high-level expression of TPL-2 with its physiological binding partners NF-κB1 p105 and ABIN-2 in HEK-293 cells generated a complex that required minimal association with chaperone proteins. This was consistent with genetic data showing that NF-κB1 p105 and ABIN-2 are essential to maintain steady-state levels of TPL-2 protein [5,7–9]. Recombinant TPL-2–ABIN-2–NF-κB1 p105 complexes could therefore be purified efficiently from transiently transfected HEK-293 cells that co-express VP35 in a non-aggregated soluble form.

### Activity and regulation of TPL-2–ABIN-2–NF-κB1 p105 complexes

Next, we tested whether TPL-2–ABIN-2–NF-κB1 p105 complex, isolated from HEK-293 cells co-expressing VP35, was catalytically active. Purified TPL-2 complex readily phosphorylated a peptide substrate corresponding to the activation loop sequence of MKK1/2 *in vitro*. Negligible catalytic activity was detected when recombinant TPL-2 complex was pre-incubated with a highly selective TPL-2 inhibitor C34 [35] or when kinase-inactive mutant TPL-2^D270A^ complex was assayed (Supplementary Figure S1 at http://www.biochemj.org/bj/452/bj4520359add.htm). The specific activity of complexed TPL-2 was not altered by VP35 co-expression (results not shown).

**Figure 4:**
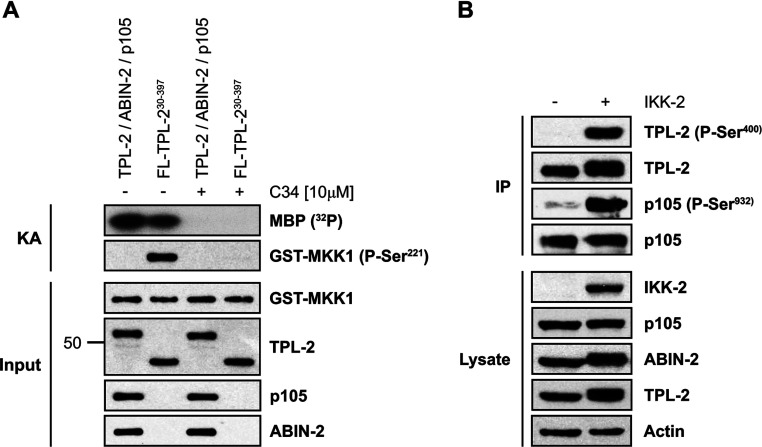
Regulation of TPL-2 complex activity and phosphorylation (**A**) *In vitro* kinase assay (KA) using purified TPL-2–ABIN-2–NF-κB1 p105 complex or FLAG–TPL-2^30–397^ as kinases (10 nM) and MBP (3.5 μM) or GST–MKK1 (0.1 μM) as substrates. MBP phosphorylation was assessed in the presence of 0.05 μCi/μl [γ-^32^P]ATP (^32^P) and analysed by autoradiography. Phosphorylation of GST–MKK1 on Ser^221^ was detected by immunoblotting. The 50 kDa band is indicated. (**B**) FLAG–TPL-2, ABIN-2–StrepII and HA–p105 were expressed transiently in VP35 co-transfected HEK-293 cells with or without 3×HA–IKK-2. At 48 h after transfection, TPL-2 complexes were immunoprecipitated (IP) with anti-FLAG antibody, and phosphorylation of TPL-2 on Ser^400^ and NF-κB1 p105 on Ser^932^ was analysed by immunoblotting. FL, FLAG.

Within cells, TPL-2 MKK1/2 kinase activity is inhibited through direct interaction with NF-κB1 p105 [5,8]. However, NF-κB1 p105 binding has no effect on the ability of TPL-2 to phosphorylate a small protein substrate, such as MBP [20]. To investigate whether TPL-2 was sensitive to NF-κB1 p105 inhibition when co-expressed with VP35, the specific activity of purified TPL-2 complex was compared with that of purified FLAG–TPL-2^30–397^, using GST–MKK1 and MBP as substrates. Although both enzyme preparations exhibited similar activity towards MBP, only FLAG–TPL-2^30–397^ phosphorylated GST–MKK1 on Ser^221^ (Figure 4A), indicating that NF-κB1 p105 regulation of TPL-2 within the purified complex was intact.

In cells, agonist activation of TPL-2 MKK1/2 kinase activity is directly regulated by IKK-2 through phosphorylation of NF-κB1 p105 on Ser^927^ and Ser^932^, which triggers NF-κB1 p105 proteasomal degradation, and direct phosphorylation of TPL-2 on Ser^400^ in its C-terminus [8,10,20,22]. Co-expression of IKK-2 with TPL-2, ABIN-2 and NF-κB1 p105 strongly induced phosphorylation of Ser^932^ in NF-κB1 p105 and Ser^400^ in TPL-2 (Figure 4B). The VP35 methodology therefore allows generation of substantial amounts of TPL-2 complex that is phosphorylated on physiological regulatory sites, which could then be used for *in vitro* functional studies.

### VP35 co-expression increases expression of other recombinant proteins

**Figure 5:**
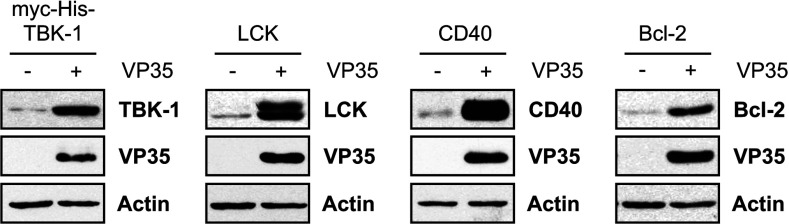
VP35 enhances expression of recombinant proteins independently of cellular localization HEK-293 cells were transiently transfected with expression plasmids encoding Myc–His–TBK1, Lck, CD40 or Bcl-2 with or without HA–VP35 or empty vector. At 48 h after transfection, lysates were analysed by immunoblotting.

To investigate whether VP35 co-expression might be used generally to boost protein production by HEK-293 cells, cells were transfected with expression constructs encoding TBK1, Lck, CD40 and Bcl-2 individually with or without VP35. Protein levels were then analysed by immunoblotting. VP35 induced substantial increases in expression of each recombinant protein (Figure 5), similar to that observed with TPL-2, ABIN-2 and NF-κB1 p105. Importantly, enhancement of expression appeared to be independent of cellular localization, since, in addition to cytoplasmic TBK1, expression of transiently expressed membrane-associated (Lck), transmembrane (CD40) and mitochondrial (Bcl-2) proteins was increased.

### Conclusions

HEK-293 cells are commonly used for expression of recombinant proteins because of their efficient transient transfection and ability to grow in suspension cultures [27,28,36–39]. However, achievable yields in HEK-293 cells are far lower than possible with *E. coli* and baculovirus expression systems [29]. The activation of PKR by transfected DNA plasmids decreases translation and limits recombinant protein production by HEK-293 cells [40]. By blocking activation of PKR, we have shown that Ebola virus VP35 substantially increased the yield of co-expressed recombinant proteins. This simple approach has potentially broad implications for structural studies and drug screening, which require large amounts of purified recombinant protein.

## Online data

Supplementary data
