# Supplementary material for: Ebola virus VP35 induces high-level production of recombinant TPL-2–ABIN-2–NF-κB1 p105 complex in co-transfected HEK-293 cells
Source: Biochem J. 2013 May 10;452(Pt 2):359–65. doi: 10.1042/BJ20121873 (PMC3727213; doi:10.1042/BJ20121873)
Supplement: Supplementary data [file bj4520359add.pdf]

Biochem. J. (2013) **452**, 359–365 (Printed in Great Britain) doi:10.1042/BJ20121873

SUPPLEMENTARY ONLINE DATA

Ebola virus VP35 induces high-level production of recombinant TPL-2–ABIN-2–NF-κB1 p105 complex in co-transfected HEK-293 cells

Thorsten GANTKE*, Sabrina BOUSSOUF*[1], Julia JANZEN*, Nicholas A. MORRICE†, Steven HOWELL‡, Elke MÜHLBERGER§ and Steven C. LEY*[2]

*Division of Immune Cell Biology, MRC National Institute for Medical Research, Mill Hill, London NW7 1AA, U.K., †Beatson Institute for Cancer Research, Bearsden, Glasgow G61 1BD, U.K., ‡Division of Molecular Structure, MRC National Institute for Medical Research, Mill Hill, London NW7 1AA, U.K., and §Department of Microbiology, School of Medicine and National Emerging Infectious Disease Laboratories, Boston University, Boston, MA 02118, U.S.A.

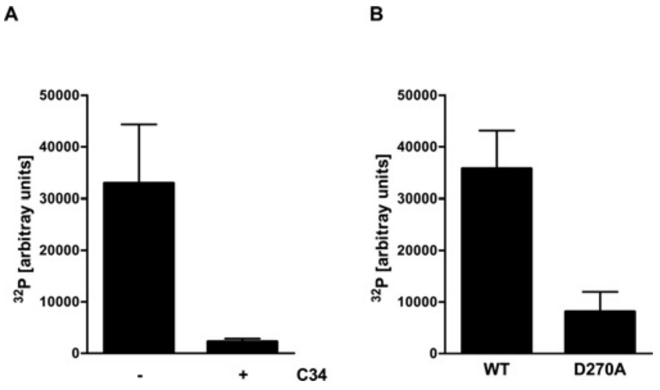

Figure S1 MKK1/2 peptide phosphorylation by purified TPL-2–ABIN-2–NF-κB1 p105 complexes

(**A**) *In vitro* kinase assay using purified wild-type (WT) TPL-2 or kinase-inactive TPL-2^{D270A} (D270A) complexes (30 nM kinase) and 50 μM MKK1/2 peptide (YAGQLIDSMANSFVGTAGKK-biotin) as substrate. Assays were performed in kinase buffer [50 mM Tris/HCl (pH 7.0), 0.03 % Brij-35, 2 mM DTT, 5 mM 2-glycerophosphate, 5 mM $MnCl_2$ and 5 % DMSO], supplemented with 0.1.mM ATP and 0.02 μCi/μl [γ-^{32}P]ATP, for 60 min at 30 °C. Reactions were stopped by the addition of EDTA, and ultrafiltrates (10 kDa) were spotted on to streptavidin-coated membranes (SAM2, Promega). Membranes were extensively washed in TBS (Tris-buffered saline)/1 % SDS, 2 M NaCl and 2 M NaCl/1 % H_3PO_4 before quantification of peptide phosphorylation by phosphorimaging. Results are means±S.D. (**B**) *In vitro* kinase assay of affinity-purified wild-type TPL-2 complex performed as in (**A**) with or without C34 TPL-2 inhibitor.

Received 14 December 2012/13 March 2013; accepted 4 April 2013
Published as BJ Immediate Publication 4 April 2013, doi:10.1042/BJ20121873

[1] Present address: GSK Medicines Research Centre, Gunnels Wood Road, Stevenage, Herts. SG1 2NY, U.K.
[2] To whom correspondence should be addressed (email sley@nimr.mrc.ac.uk).

© 2013 The Author(s)
The author(s) has paid for this article to be freely available under the terms of the Creative Commons Attribution Licence (CC-BY) (http://creativecommons.org/licenses/by/3.0/) which permits unrestricted use, distribution and reproduction in any medium, provided the original work is properly cited.

Biochemical Journal www.biochemj.org
